# Patient-Specific Modeling of Regional Antibiotic Concentration Levels in Airways of Patients with Cystic Fibrosis: Are We Dosing High Enough?

**DOI:** 10.1371/journal.pone.0118454

**Published:** 2015-03-03

**Authors:** Aukje C. Bos, Cedric van Holsbeke, Jan W. de Backer, Mireille van Westreenen, Hettie M. Janssens, Wim G. Vos, Harm A. W. M. Tiddens

**Affiliations:** 1 Department of Pediatric Pulmonology, Erasmus Medical Centre-Sophia Children’s Hospital, Rotterdam, the Netherlands; 2 Department of Radiology, Erasmus Medical Centre, Rotterdam, the Netherlands; 3 FluidDA nv, Kontich, Belgium; 4 Department of Medical Microbiology and Infectious Diseases, Erasmus Medical Centre, Rotterdam, the Netherlands; University Children’s Hospital Basel, SWITZERLAND

## Abstract

**Background:**

*Pseudomonas aeruginosa (Pa)* infection is an important contributor to the progression of cystic fibrosis (CF) lung disease. The cornerstone treatment for *Pa* infection is the use of inhaled antibiotics. However, there is substantial lung disease heterogeneity within and between patients that likely impacts deposition patterns of inhaled antibiotics. Therefore, this may result in airways below the minimal inhibitory concentration of the inhaled agent. Very little is known about antibiotic concentrations in small airways, in particular the effect of structural lung abnormalities. We therefore aimed to develop a patient-specific airway model to predict concentrations of inhaled antibiotics and to study the impact of structural lung changes and breathing profile on local concentrations in airways of patients with CF.

**Methods:**

In- and expiratory CT-scans of children with CF (5–17 years) were scored (CF-CT score), segmented and reconstructed into 3D airway models. Computational fluid dynamic (CFD) simulations were performed on 40 airway models to predict local Aztreonam lysine for inhalation (AZLI) concentrations. Patient-specific lobar flow distribution and nebulization of 75 mg AZLI through a digital Pari eFlow model with mass median aerodynamic diameter range were used at the inlet of the airway model. AZLI concentrations for central and small airways were computed for different breathing patterns and airway surface liquid thicknesses.

**Results:**

In most simulated conditions, concentrations in both central and small airways were well above the minimal inhibitory concentration. However, small airways in more diseased lobes were likely to receive suboptimal AZLI. Structural lung disease and increased tidal volumes, respiratory rates and larger particle sizes greatly reduced small airway concentrations.

**Conclusions:**

CFD modeling showed that concentrations of inhaled antibiotic delivered to the small airways are highly patient specific and vary throughout the bronchial tree. These results suggest that anti-*Pa* treatment of especially the small airways can be improved.

## Introduction

Cystic fibrosis (CF) is a severe hereditary and life-threatening disease in the Caucasian population. Most morbidity and mortality (>90% of deaths) is caused by progressive lung disease [1]. Important components of the pathophysiology of CF lung disease are bronchiectasis, small airways disease [2–5] and chronic infection with *Pseudomonas aeruginosa* (*Pa*) as the main pathogen [1].

Inhaled antibiotics play a central role in eradication and chronic suppressive therapy of *Pa* infections. Unfortunately, despite these interventions, lung disease in CF eventually progresses to end-stage, with substantial small airways disease in most patients [6]. Significant mucus accumulation and wall thickening in the small airways has been found in explant CF lungs with end-stage lung disease [7, 8] and this has been associated with the presence of *Pa* [9]. Hence, more effective anti-*Pa* therapies, especially those targeted at the small airways, may offer an opportunity to improve patient outcomes.

The generally held view that inhaled antibiotics result in high concentrations within the airways is largely based on high drug concentrations found in sputum [10, 11]. However, it is unlikely that sputum concentrations are representative for the small airway concentrations. The drug reaching the small airways is distributed over a much larger surface area, namely a 30–190-fold greater area compared to central airways [12]. In addition, mucociliary transport clears sputum from the small airways via the central airways, taking up additional drug during transit, before expectoration. Thus, the final sputum concentration is likely to overestimate small airway concentration.

Very little is known about antibiotic concentrations in the small airways, due to the difficulty of *in vivo* measurement. The progression of small airways disease despite anti-*Pa* treatment suggests that small airway deposition of inhaled antibiotics may be insufficient. To optimize *Pa* eradication and chronic suppression with inhaled antibiotics, it is important to obtain local concentrations equal to or above the minimal inhibitory concentration (MIC). Concentrations below the MIC lead to the development of *Pa* strains with high mutation rates, and hence resistant subpopulations of *Pa* which cannot be eradicated [13].

Extensive research has been done to understand aerosol deposition mechanisms. It has been well established that aerosol deposition is strongly dependent on particle size [14], airflow, inhalation technique, lung structural changes and airway obstruction by mucus [15]. CF-patients with more severe lung disease have more central airway deposition compared to healthy individuals [15]. This suggests that dose adjustments and particle size optimization, or inhalation technique, could improve aerosol delivery to the site of infection. However, to maximize drug delivery to the small airways, the impact of age, structural changes and inspiratory flow profile on antibiotic concentrations in different compartments of the bronchial tree needs to be better understood.

Unfortunately, it is difficult to investigate the simultaneous influence of the above-mentioned factors on deposition *in vivo*. An *in silico*, patient-specific model based on computational fluid dynamics (CFD) has been developed to assess the behavior of inhalation medication in airways [16]. This technique has been validated using Single Photon Emission Computed Tomography [17]. To date, this technique has been used to study lung drug deposition in asthma [18], to assess airflow distribution in both asthma and chronic obstructive pulmonary disease [16, 17], and the bronchodilating effects of β2-agonists [19–21].

In CF, CFD can allow us to study the relation between airway morphology and local concentrations of inhaled antibiotics. Additionally, by repeating simulations with various model parameters, CFD can provide more information on how to optimize small airway aerosol deposition in CF-patients with structural lung changes.

This is the first study using patient-specific airway models with varying disease severity and CFD to estimate aerosol concentrations in both the central and small airways of patients with CF. We aimed to study the relation between structural lung disease and deposition of an inhaled antibiotic used for suppressive treatment of chronic *Pa* infections, Aztreonam lysine for inhalation (AZLI; Gilead Pharmaceuticals, Foster City, USA). AZLI is a monobactam antibiotic, delivered by the e-Flow electronic nebulizer [11]. We hypothesized that:

a) there is great variation in AZLI concentrations between patients, due to differences in airway geometry and lung disease severity,
b) AZLI concentrations in the small airways would be below the MIC for *Pa* in patients with more severe lung disease, and
c) AZLI concentrations in the small airways could be improved by increasing the dose of AZLI or by modifying the inhalation technique.

## Materials and Methods

### Study population

We included all spirometer controlled volumetric in- and expiratory high resolution CT-scans, with a slice thickness of 1 mm or less, performed as part of the routine annual CF check-up in the CF-centre of Erasmus MC-Sophia Children’s Hospital (Rotterdam, Netherlands) between 2008 and 2012 (aged 5–17 years). Patients were diagnosed with CF by a positive sweat test and/or genotyping for known CF mutations. Demographic data and pulmonary function tests were collected prior to the CT-scan. Pulmonary function test results were expressed as percentages of predictive values, according to Stanojevic for the forced vital capacity (FVC) and forced expiratory volume in 1 second (FEV_1_), and Zapletal for the forced expiratory flow at 75% (FEF_75_) [22, 23]. Written informed consent for the use of de-identified data was obtained from the parent/guardian and subjects ≥ 12 years. This retrospective study was approved by the Institutional Review Board of the Erasmus Medical Center in Rotterdam, the Netherlands (MEC-2013–078).

### Chest Computed Tomography (CT)

Forty spirometer controlled CT-scans of consecutive CF-patients, acquired as part of routine clinical care, were included. To quantify chest CT abnormalities, we used the validated CF-CT scoring system [24]. The lobar specific CF-CT score per component was used and expressed as a percentage of the maximum possible score per lobe. The component scores for bronchiectasis, airway wall thickening and air trapping were used for analysis. Detailed descriptions of CT scanning protocol and CT evaluation are available in the supporting information of this paper (S1 Text).

### Reconstruction of three-dimensional airway models

Based on the inspiratory scan, a semi-automatic algorithm was used to reconstruct a patient-specific three-dimensional (3D) model of the intra-thoracic region. This intra-thoracic region was defined arbitrarily as the lower airway. Automatic airway segmentation was performed up to the point where no distinction could be made between the intra-luminal and alveolar air. Following automated segmentation of the bronchial tree, the airways were manually checked. Missing branches were added to the bronchial tree and incorrect branches were deleted when necessary; 3.39±2.51% of the branches needed to be manually altered. The respiratory tract was reconstructed down to the level of airways with a diameter of 1–2mm. The segmented airway tree was converted into a 3D model that was smoothed using a volume compensation algorithm. The smoothed model was trimmed perpendicular to the airway centreline at the trachea (using the middle point of the superior side of the sternum as a landmark) and at each terminal bronchus. Remaining artefacts due to noise in the CTs were then manually removed from the model.

For the upper (extra-thoracic) airways, a generic average adult upper airway model was selected and scaled down in such a way that both the anteroposterior and lateral dimension of the scaled model’s trachea, at the location of the sternum, matched the average anteroposterior (1.25cm) and lateral (1.19cm) dimension for the 40 patients. The upper airway model was connected with a reverse engineered mouthpiece of the Pari eFlow. Reverse engineering was done based on a CT-scan of the mouthpiece taken on a GE LightSpeed VCT (80kV, 18.25mAs, 0.311mm slice increment, 0.188mm pixel size, STANDARD reconstruction algorithm). The mouthpiece/upper airway model was trimmed perpendicular to the centreline of the trachea (again using the middle point of the superior side of the sternum as a landmark). This ensured correct positioning of the upper airway model with respect to the patient-specific airway models. Models were then coupled using the freeform hole filling algorithm of 3-Matic.

For each of the 40 CT-scan sets, the patient-specific lower airway model was connected to the selected nebulizer mouthpiece/upper airway model, maximizing the contribution of patient-specific information. All segmentation and 3D model operations were performed in commercially available validated software packages (Mimics 15.0 and 3-Matic 7.0, Materialise N.V., Belgium, Food and Drug Administration, K073468; Conformité Européenne certificate, BE 05/1191.CE.01).

### Meshing

The triangulated, mouthpiece/upper/lower airway surface models had a maximum triangle edge length of 0.5 mm and a minimum triangle aspect ratio of 0.4. These models were converted to tetrahedral 3D volume meshes using TGrid 14.0 (Ansys Inc, Canonsburg, PA). A boundary layer with a growth rate of 1.4 was included in the models. Maximal tetrahedral volume was set to 2 mm^3^ and maximal equilateral volume-based skewness to 0.9. Grid convergence demonstrated that a mesh size of 2.9±0.7M [1.9–4.6] cells is appropriate for the study, depending on the size of the patient-specific lower airway model. Meshing was done on 1 CPU and meshing time was below 200s.

### Reconstruction of three-dimensional lung lobes

From both the inspiratory and expiratory CT-scans, the patient-specific lung lobes were extracted using a semi-automated tool that identifies the fissures separating the lung lobes. The internal lobar flow distribution was calculated based on the lobar volume change from expiration to inspiration. Lung lobe identification has been performed in a commercially available validated software package (Mimics 15.0, Materialise N.V., Belgium, Food and Drug Administration, K073468; Conformité Européenne certificate, BE 05/1191.CE.01).

### Inlet of the airway model

#### Breathing profile

The median age of the patient population (11 years) was used to generate a generic breathing profile based on the following parameters: the median weight of 11 year old Dutch children is 38 kg (boy: 37 kg, girl 38.5 kg) [25]; tidal volume of 10 ml/kg (380 ml); respiration rate (18 breaths per minute) [26]. The resulting profile had an inspiration/expiration ratio of 1:2 and a sinusoidal shape, see S1 Fig.

To be able to examine the flow dependency of the simulated results, two additional breathing profiles were generated: (1) a high breathing profile, consisting of a higher tidal volume of 14 ml/kg (532 ml) and the respiratory rate of the youngest age (5 years: 22 breaths per minute), and (2) a low breathing profile, consisting of a lower tidal volume of 6 ml/kg (228 ml) and the respiratory rate of oldest age (17 years: 14 breaths per minute). These additional profiles can also be found in S1 Fig.

#### Aerosol characteristics

Eleven different trials (Anderson Cascade impactor n = 6, next generation impactor n = 2, and laser diffraction n = 3) studied the diameter distribution of AZLI nebulized via the Pari eFlow (Gilead data on file). The extremes and the median of these unpublished trials were selected for use in the CFD simulations: smallest diameter (2.81±1.47 μm), median diameter (3.18±1.63μm) and largest diameter (4.35±2.05 μm). Furthermore, an *in vivo* characterization of the eFlow showed that 35% of the nominal fill volume is either trapped in the mouthpiece or exhaled.

#### Flow simulation

Computational fluid dynamics (CFD) flow simulations were performed in Fluent 14.0 (Ansys Inc, Canonsburg, PA). Drug release in the simulated nebulizer was continuous. Therefore, particles were injected during the whole breathing cycle. All simulations were transient using a second order time-stepping algorithm and a time-step of 0.005s. Turbulence was evaluated through large eddy simulations with a turbulent kinetic subgrid model. Aerosol transport was modeled by an implicit Runge-Kutta Lagrangian discrete particle model, with a one-way coupling of the forces from the flow to the particle and taken into account the Saffman lift forces. Transient particle tracking was used and the particle time-step was equal to the flow time-step. Every time-step, 15862 particles were injected. This number is based on particle convergence studies. Particles were considered deposited the moment they hit the airway wall.

The nominal dose of 75 mg AZLI was corrected for the 35% combined inhaler loss and exhaled fraction (Gilead data on file). Due to the incorporation of the exhaled fraction, only the inhalation was modelled. The boundary condition at the inhaler mouthpiece was represented by the inhalation part of the mean breathing profile in S1 Fig. The downstream boundary conditions at the terminal bronchi were set such that the percentage of flow exiting the model towards a lobe did match with the internal lobar flow distribution obtained from the expiratory and inspiratory CT data.

To investigate the influence of the inhalation manoeuvre on local concentrations, additional simulations were performed in a subset of the population (2 tallest, 2 smallest, 2 median sized patients). These additional CFD simulations were performed with the altered breathing profiles described in Section: ‘Breathing profiles’.

### Calculation of regional AZLI concentrations

#### Aerosol deposition analyses

**Fig 1:**
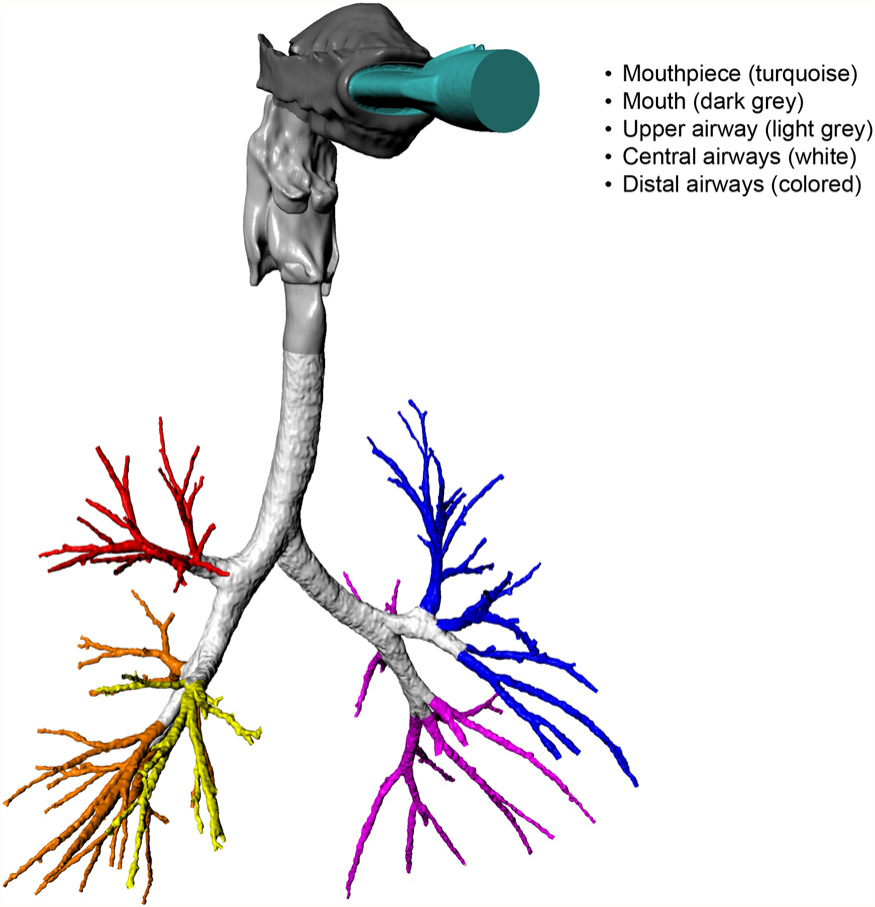
Coupled mouthpiece/upper/lower airway model. Coupled mouthpiece/upper/lower airway model subdivided in multiple regions. Airways are segmented up to the 5^th^-9^th^ generation.

To be able to perform regional analyses, the respiratory tract was subdivided into multiple regions. For the airways with a diameter >1–2 mm these regions were obtained from the mouthpiece/upper/lower airway model, see Fig. 1. In this figure the upper airway is divided in two parts: the oral cavity and the pharynx; and the lower airways are divided into central part and distal parts representing the lung segments. Conducting airways with a diameter <1–2 mm could not be distinguished from the CT images and have been added to the patient-specific model by using Phalen’s description of the airway tree in infants, children and adolescents[12]. For every simulation, a Phalen model was constructed based on the height of the specific patient.

Regional AZLI deposition was evaluated for both the particles depositing inside the model, in every separate zone indicated in Fig. 1; as well as for the particles exiting the model at the terminal bronchi, in the small airways represented by the Phalen model on a lobar basis. Once the aerosol entered the Phalen model of a certain lobe, it was assumed that it was distributed homogeneously.

#### Airway surface liquid

To compute AZLI concentrations in the airway surface liquid (ASL) throughout the bronchial tree we used a range of thicknesses based on studies in CF. Three different ASL scenarios were considered: thick ASL (7 μm) [27], thin ASL (3 μm) [28] and the mean ASL (5 μm).

#### AZLI concentrations

For each reconstructed airway and for each lung lobe, the area was calculated and the CFD simulations provided data on the drug deposition in that region. The regional AZLI concentration was computed as follows: the mass of the deposited drug in an airway was divided by the thickness of the lining fluid multiplied by the surface area of that airway.

Since the flow simulations were performed with 3 different sizes for aerosol diameter and the AZLI concentrations were calculated using 3 different thicknesses for ASL, this resulted in 9 different scenarios for which we calculated the concentrations: a scenario with the smallest diameter and smallest ASL thickness, scenario with smallest diameter and median ASL thickness and so on.

Finally, the regional AZLI concentration is expressed relative to the MIC of AZLI for *Pa*. The accurate AZLI concentration for effective killing of *Pa* in an *in vivo* CF lung is not well-defined. Studies on the efficacy of AZLI mostly use a threshold of 10-fold MIC_90_ [29, 30], which describes the MIC required to inhibit the growth of 90% of *Pa* strains multiplied by 10. This threshold was used in this study, combined with the highest reported MIC_90_ value in literature: 128 μg/ml [13]. This MIC_90_ value refers to all *Pa* isolates, both non-mucoid and mucoid, as well as strains with and without resistance mechanisms. Thus we expressed the regional AZLI concentration directly after inhalation relative to 10x128 μg/ml = 1280 μg/ml. With this stringent effective AZLI level we took into account the mix of *Pa* populations within one patient with variability in geno- and phenotypes of strains including resistant subpopulations [13], and clearance of drug starting directly after nebulization.

### Statistical analysis

Inter- and intra-observer agreements of CF-CT subscores were calculated using intraclass correlation coefficients (ICC). Although no universally accepted standards are available for what constitutes good reliability, ICC values between 0.4 and 0.6, 0.6 and 0.8, and ≥ 0.8 are generally considered to represent moderate, good and very good agreement, respectively. Systematic errors in component scores were evaluated using Bland-Altman plots, expressing the differences between two observers as a function of their mean [31].

To establish the correlation between age and disease severity expressed in CF-CT scores and pulmonary function tests, we used Spearman’s correlation test. According to Cohen’s criteria (1988), correlations between 0.10 and 0.29 are considered weak, between 0.30 and 0.49 moderate and above 0.50 are considered strong.

Differences between multiple groups were investigated using a Kruskal-Wallis test, after which two-by-two comparisons were made using Mann-Whitney tests. The effect size was noted as “r”. Correlations between parameters measured in different lobes were studied using a generalized estimating equation with an autoregressive covariance matrix to account for within-subject correlations. All data are presented as median (range). Significance level was set at 0.05 and p-values were corrected for multiple testing using Benjamini and Hochberg correction [32]. All statistical computations were performed using the open-source statistical environment R 2.15.3.

## Results

### Study population

Forty inspiratory and expiratory chest CT-scans were selected from 31 patients. Baseline characteristics are shown in Table 1. Thirty-nine (98%) CT-scans were spirometer controlled; the remaining scan was performed with technician guidance.

**Table 1:** Baseline characteristics.

	Value	
N	31	
- Nr of patients with 1 CT	22	
- Nr of patients with 2 CTs	9	
Male	11	35%
Age	11.0	5.8–17.3
Bronchiectasis score (% of max CF-CT score)	2.8	0.0–16.0
Airway wall thickening score (% of max CF-CT score)	3.7	0.0–18.5
Air trapping score (% of max CF-CT score)	22.2	11.1–85.2
FEV_1_%pred	94.2	70.8–115.4
FVC %pred	104.3	78.7–127.9

Data are presented as nr. (%) or median (range), unless otherwise indicated.

There were no significant differences between the sexes for demographics, pulmonary function tests and CF-CT subscores, therefore the dataset did not have to be split in sex groups.

ICCs for within-observer agreement ranged from 0.85 (air trapping) to 0.93 (bronchiectasis), whereas between-observer agreement ranged from 0.67 (airway wall thickening) to 0.77 (air trapping).

### Correlations with age

There was a moderate positive correlation between bronchiectasis and age (r_s_ = 0.481, p = 0.005). Correlations between age and airway wall thickness (r_s_ = 0.302, p = 0.087), air trapping (r_s_ = 0.096, p = 0.554) and pulmonary function tests (FVC% pred: r_s_ = 0.053, p = 0.885; FEV1% pred: r_s_ = -0.024, p = 0.885) were not significant.

### Deposition analyses

The software tool used to identify the boundaries between the lobes in the lungs, i.e. the pulmonary fissures, could not identify the fissure between the right upper and middle lung lobes in 18 patients and between the left upper and lower lung lobes in 1 patient. These lung lobes were excluded for analysis of AZLI deposition.

**Fig 2:**
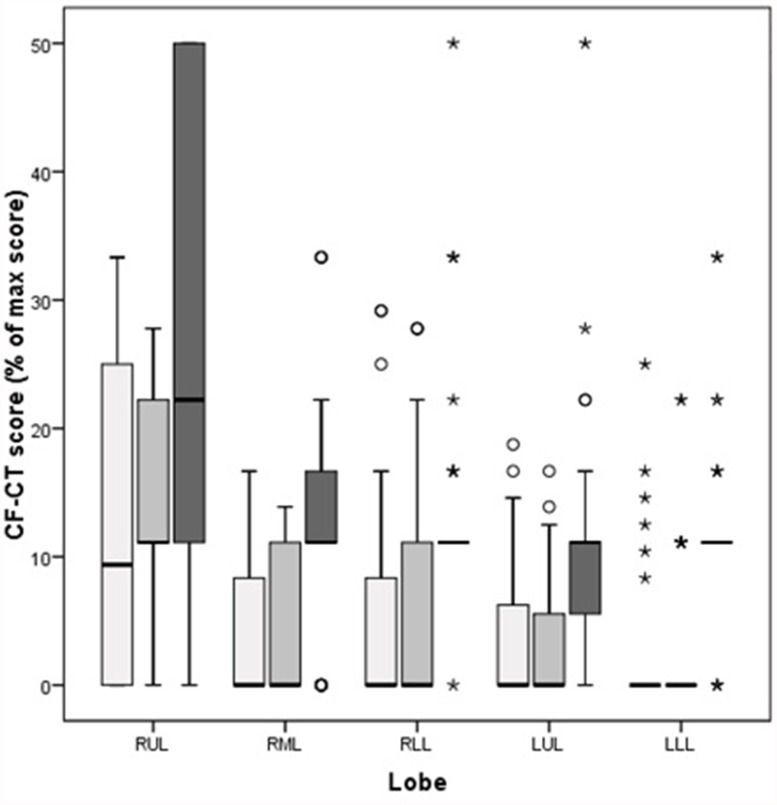
Comparison of CF-CT subscores per lobe. Comparison of CF-CT subscores per lobe, presented as % of max CF-CT score. Data are presented as median (range), unless otherwise indicated. White bars represent bronchiectasis score, light grey bars represent airway wall thickening score and dark grey bars represent air trapping score. RUL = right upper lobe (n = 22), RML = right middle lobe (n = 22), RLL = right lower lobe (n = 40), LUL = left upper lobe (n = 39), LLL = left lower lobe (n = 39).

Significant differences between the lobes were found for all tested CF-CT subscores (bronchiectasis: x^2^ = 21.70, p<0.001; airway wall thickness: x^2^ = 25.22, p<0.001; and air trapping: x^2^ = 20.15, p<0.001). It was found that CF-CT subscores were generally higher in the right upper lobe than in the other lobes (Fig. 2).

**Fig 3:**
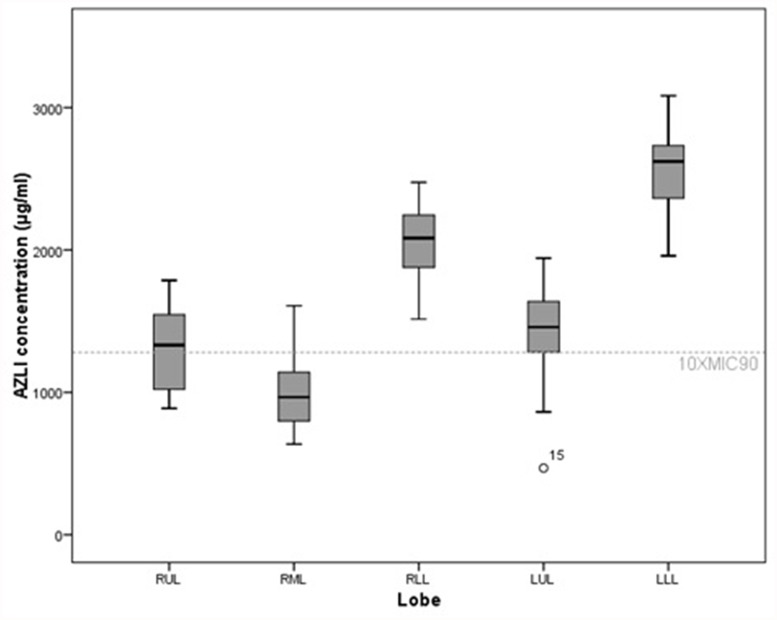
Differences between lobes in AZLI concentrations. Differences between lobes in AZLI concentrations for the scenario of thick airway surface liquid with largest aerosol diameter. Data are presented as median (range), unless otherwise indicated. Significant differences in AZLI concentrations were found between all lobes, except for one pairwise comparison (see S1 Table). RUL = right upper lobe, RML = right middle lobe. RLL = right lower lobe, LUL = left upper lobe, LLL = left lower lobe.

There were differences in AZLI deposition between the different lobes (Fig. 3). The highest AZLI concentrations were found in the lower lobes. For the lower lobes, AZLI concentrations were always above 10xMIC_90_ independent of the scenario tested. An inverse correlation between AZLI concentration in a lobe and the CF-CT scores was observed, indicating that more diseased lobes received less drug (Table 2). For example, when assuming small diameters and thin lining fluids, a reduction in AZLI concentration of 439 μg/ml was observed for every 1% of point increase in bronchiectasis score.

**Table 2:** Inverse correlation between AZLI concentration in a lobe and the CF-CT scores.

ASL	Diameter (μm)	CF-CT score	Estimate	Std.err	Wald	p-value
Thin (3 μm)
	Smallest (2.9)	Bronchiectasis	-439	115	14.6	**0.00013**
		Airway wall thickness	-466	142	10.7	**0.0011**
		Air trapping	-237.5	85.3	7.76	**0.0053**
	Median (3.18)	Bronchiectasis	-387	108	12.8	**0.00034**
		Airway wall thickness	-421	133	10	**0.0016**
		Air trapping	-229.7	78.5	8.56	**0.0034**
	Largest (4.35)	Bronchiectasis	-278.8	86.5	10.4	**0.0013**
		Airway wall thickness	-316.8	106.2	8.9	**0.0029**
		Air trapping	-190.3	61.4	9.6	**0.0019**
Median (5 μm)
	Smallest (2.9)	Bronchiectasis	-263.7	69	14.6	**0.00013**
		Airway wall thickness	-279.6	85.4	10.7	**0.0011**
		Air trapping	-142.5	51.2	7.76	**0.0053**
	Median (3.18)	Bronchiectasis	-232	64.8	12.8	**0.00034**
		Airway wall thickness	-252.7	79.9	10	**0.0016**
		Air trapping	-137.8	47.1	8.56	**0.0034**
	Largest (4.35)	Bronchiectasis	-167.3	51.9	10.4	**0.0013**
		Airway wall thickness	-190.1	63.7	8.9	**0.0029**
		Air trapping	-114.2	36.9	9.6	**0.0019**
Thick (7 μm)
	Smallest (2.9)	Bronchiectasis	-188.3	49.3	14.6	**0.00013**
		Airway wall thickness	-199.7	61	10.7	**0.0011**
		Air trapping	-101.8	36.5	7.76	**0.0053**
	Median (3.18)	Bronchiectasis	-165.7	46.3	12.8	**0.00034**
		Airway wall thickness	-180.5	57.1	10	**0.0016**
		Air trapping	-98.5	33.7	8.56	**0.0034**
	Largest (4.35)	Bronchiectasis	-119.5	37.1	10.4	**0.0013**
		Airway wall thickness	-135.8	45.5	8.9	**0.0029**
		Air trapping	-81.6	26.3	9.6	**0.0019**

Inverse correlation between AZLI concentration in a lobe and the CF-CT scores, shown for the different scenarios and different aerosol diameters. For example: for every 1% of point increase in bronchiectasis score, a reduction in AZLI concentration of 439 μg/ml is observed assuming small diameters and thin lining fluids. Number of lobes used for analysis: right upper lobe (n = 22), right middle lobe (n = 22), right lower lobe (n = 40), left upper lobe (n = 39), left lower lobe (n = 39). P-values in bold represent significant differences. ASL = airway surface liquid.

**Fig 4:**
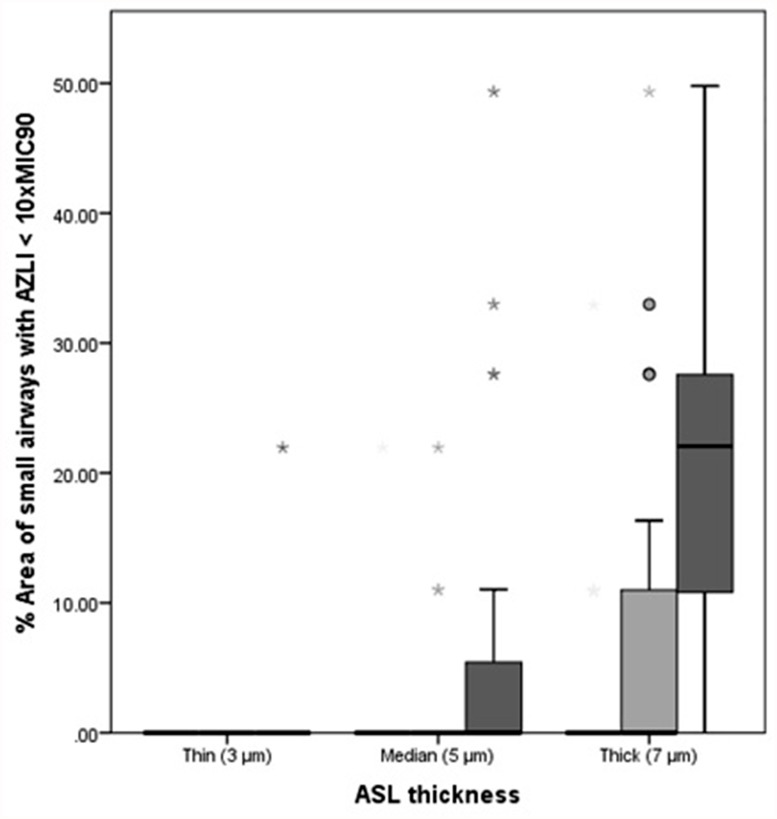
Percentage area of small airways with AZLI <10xMIC_90_. Percentage area of small airways with AZLI concentrations <10xMIC_90_. Data are presented as median (range) for the different scenarios. White bars represent the smallest aerosol diameter (2.9 μm), light grey bars represent the median aerosol diameter (3.18 μm) and dark grey bars represent the largest aerosol diameter (4.35 μm). ASL = airway surface liquid.

**Fig 5:**
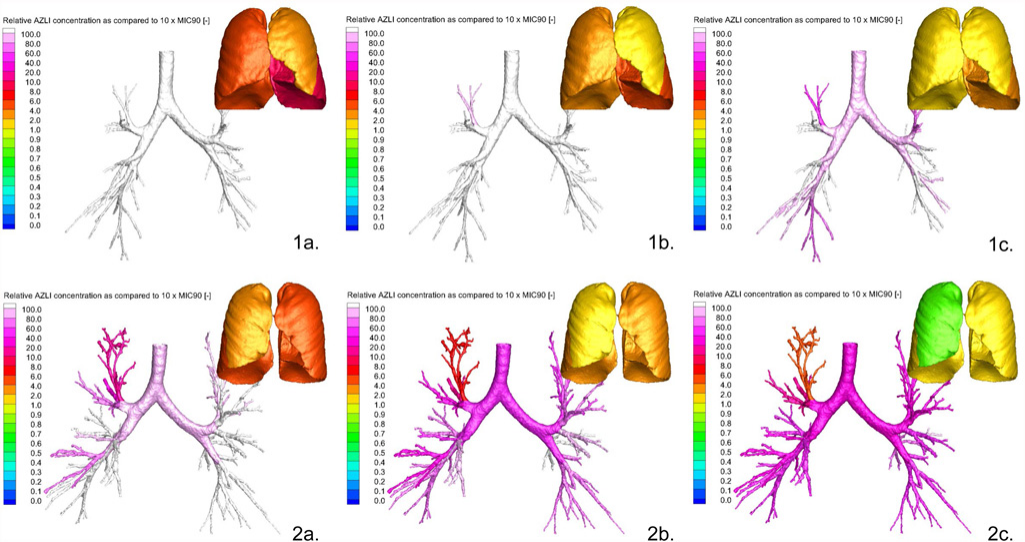
Relative AZLI concentrations in central and small airways of 2 patients for 3 different scenarios. Simulations of AZLI deposition in 2 patients, representing 3 scenarios of varied airway surface liquid thickness (ASL) and aerosol diameter. Severity of CF lung disease was determined by the CF-CT score (% of total CF-CT score). Scenario a = thin ASL with smallest aerosol diameter; scenario b = median ASL with median aerosol diameter; scenario c = thick ASL with largest aerosol diameter. Part 1a, 1b and 1c: Patient 1, mild CF lung disease: bronchiectasis 0.0%, airway wall thickening 0.0% and air trapping 11.1%. Patient 1 received concentrations > 10xMIC_90_ in the central and small airways independent of ASL thickness and aerosol diameter (Part 1a, 1b, 1c). Part 2a, 2c and 2c: Patient 2, more severe lung disease: bronchiectasis 12.5%, airway wall thickening 11.1% and air trapping 38.9%. Patient 2 received concentrations > 10xMIC_90_ in the central and small airways in scenario a and b (Part 2a and 2b), but AZLI concentrations < 10xMIC_90_ in the small airways in scenario c (right upper and middle lobes) (Part 2c).

AZLI concentrations were calculated with 3 different ASL thicknesses. Because of the formula used for calculations, the ASL thickness was of direct influence on the concentrations: the thicker the ASL the lower the AZLI concentration (S2 Fig.). Therefore, when expressing this regional AZLI concentration relative to 10xMIC_90_, the thicker the ASL the larger the area of small airways with AZLI concentrations below 10xMIC_90_. The combination of thin ASL and smallest aerosol diameter resulted in AZLI concentrations above 10xMIC_90_ for both large and small airways. For the combination of thick ASL and largest aerosol diameter, 22% (0–49.79%) of the total area of small airways received AZLI concentrations below 10xMIC_90_. The lowest AZLI value observed in the small airways for the tested population was 468.14 μg/ml or 3.66xMIC_90_. Fig. 4 summarizes the percentage of area of small airways that receive a concentration below 10xMIC_90_ for the different modeling conditions. In Fig. 5, the relative AZLI concentrations in 2 patients are shown for 3 different scenarios. In the central and distal airways, AZLI concentrations 10–100x above the threshold of 1280 μg/ml were observed. In the small airways (visualized per lobe in the images), lower concentrations were seen.

**Fig 6:**
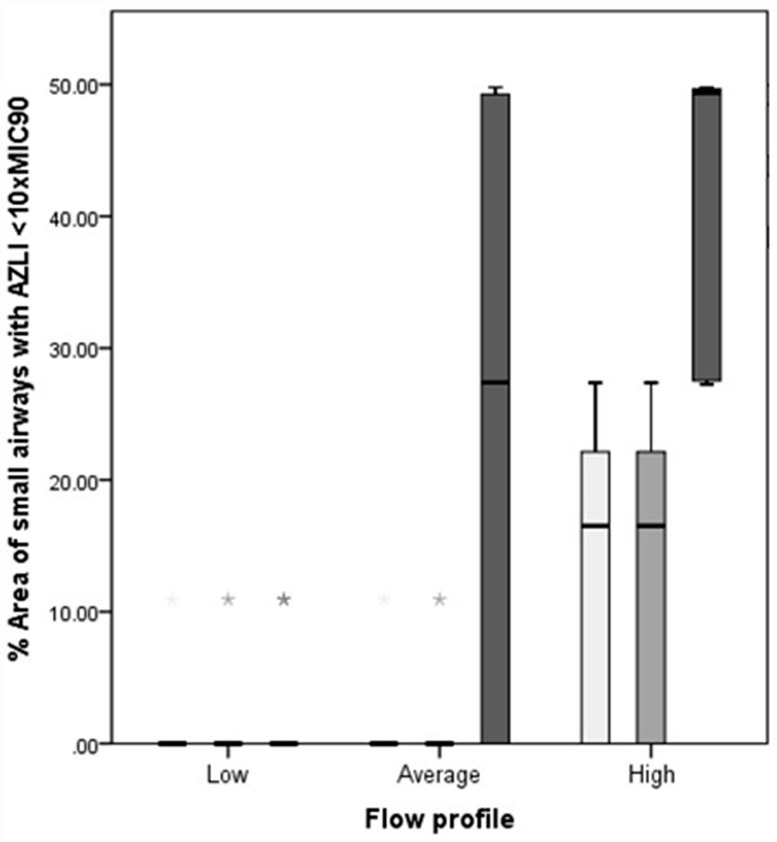
Influence of inhalation technique on AZLI concentrations. Influence of inhalation technique on AZLI concentrations presented as percentage area of small airways with AZLI <10xMIC_90_. Low breathing profile: tidal volume of 6 ml/kg (228 ml) and respiration rate of 14 breaths/min. Average breathing profile: tidal volume of 10 ml/kg (380 ml) and respiration rate of 18 breaths/min. High breathing profile: tidal volume of 14 ml/kg (532 ml) and respiration rate of 22 breaths/min. Data are presented as median (range) for the different scenarios. Light grey bars represent the scenario of median ASL (5 μm) with largest aerosol diameter (4.35 μm). The darker grey bars represent the scenario of thick ASL (7 μm) with median aerosol diameter (3.18 μm) and the darkest grey bars represent the scenario of thick ASL (7 μm) with largest aerosol diameter (4.35 μm). The scenarios of thin ASL with all diameters, median ASL with smallest and median diameter and thick ASL with smallest diameter are not represented as all breathing profiles resulted in AZLI concentrations above 10xMIC_90_. ASL = airway surface liquid.

Decreasing the tidal volume and respiratory rates decreased the deposition in the extra-thoracic region (mouth and upper airway), subsequently resulting in significantly less areas with a concentration below 10xMIC_90_ in the lungs (Fig. 6).

## Discussion

To our knowledge this is the first study that used CFD to estimate patient-specific inhaled antibiotic concentrations throughout the bronchial tree in CF. The most important finding was that the airway concentrations were highly dependent on patient-related factors.

Another important finding of this study was that effective AZLI concentrations above the 10xMIC_90_ threshold for *Pa* were observed throughout the lung in most simulated conditions. However, variables such as particle diameter and ASL thickness had a significant impact on the results. Under certain values of these variables, it was shown that the concentration would drop below 10xMIC_90_ in 22% of the small airways area. The most critical scenario was the combination of a thick ASL and the largest aerosol diameter. However, the lowest observed AZLI concentration in the small airways of the studied population was still above 3xMIC_90_ for *Pa*. For this particular patient, an increase of 2.7 times the standard nebulized AZLI dose would have resulted in sufficiently high concentrations in the small airways. In the ‘best-case’ scenario, both large and small airways received AZLI concentrations above 10xMIC_90_. The observation that regional low concentrations can exist is of great importance, since suboptimal concentrations could result in insufficient killing and are associated with increases in mutation frequencies [33]. These hypermutator strains are resistant against antimicrobials used in CF and hence, rather than elimination of the pathogen, treatment will result in even further selection of these resistant subpopulations [13].

As in previous studies we observed that the upper lobes were more severely affected by structural disease relative to the other lobes [5]. The reason for this distribution is still largely unknown, however our findings suggest that uneven distribution of inhaled drugs could contribute to this inequality. We observed that even in patients with relatively little structural damage, the upper lobes received lower AZLI concentrations than the lower lobes.

We found an inverse correlation between lobar CF-CT score and AZLI concentration. The population included in this study had early to moderately advanced lung disease on CT, with well-preserved lung function. Patients with more advanced lung disease would be further affected by the uneven distribution of inhaled drugs. These findings match deposition studies in patients with CF, showing that the deposition pattern is more heterogeneous in diseased lungs than in healthy lungs [14, 15]. In addition, it supports previous studies showing that penetration of inhaled drugs in deformed or partially obstructed airways is restricted [15]. These results suggest that upper lobes are more vulnerable to under-treatment and that this effect is stronger once structural damage is present.

With our simulations, we showed that lower inhalation flows reduced extra-thoracic deposition, leading to higher AZLI concentrations in the small airways. This finding is consistent with previous studies showing that high flows lead to high extra-thoracic and upper airway drug deposition [14]. Using patient-specific airway modeling, we were able to study the impact of inhalation flow rate and inhaled volume on local airway drug concentrations in the small airways. This information can be used to design smart nebulizers in such a way that adequate small airway concentrations can be obtained [34], or to define the required medication dose for a patient that, independent of the breathing pattern, results in sufficient drug delivery to critical areas of the lung.

CFD offers a number of advantages that complement available techniques for studying aerosol deposition. Non-imaging techniques, e.g. pharmacokinetic methods, lack the ability to identify dose deposition into different zones of the lungs [35]. Scintigraphic methods do assess the deposition location of inhaled drugs, however, by dividing the lung into several large regions of interests [36]. 3-helium MRI provides structural information and offers a quantification of ventilation down to the alveolar level [37], however regional deposition of inhaled drugs cannot be derived from this technique. In contrast, CFD allows detailed information on aerosol deposition at specific anatomical sites to be determined. Another advantage is that CFD allows estimation of AZLI concentrations throughout the bronchial tree, data that are extremely difficult to obtain *in vivo*. To date, great emphasis has been given to sputum concentrations in clinical studies investigating inhaled antibiotics. It is highly likely that these concentrations are primarily reflective of central airway concentrations. The central airway concentrations found in this study were in the range of the fitted sputum concentrations from clinical studies, taking into account that published sputum samples were collected at later time-points compared to this study (data on file). As suggested by our findings, higher concentrations in central airways result in lower concentrations in small airways, challenging the validity of sputum samples as a useful indicator to explain the failure or success of inhaled antibiotics. We utilized CT-scans that were acquired as part of routine clinical care [38], allowing extraction of extra clinical relevant information without the need for additional radiation. Unlike other deposition study techniques, our model allowed us to study the impact of multiple variables, e.g. differences in particle size, on lung deposition within the same patient. CFD can therefore be used to predict lung deposition and effective dose of newly developed nebulizers. CFD opens up new pathways to further optimize inhalation therapies, even at a personalized level.

Our modeling study has a number of limitations. To allow modeling of AZLI and estimation of concentrations, several assumptions were made. The first assumption was that the antibiotic concentration in the ASL is the most important determinant for effective killing of *Pa* [39–41]. To estimate ASL concentrations we had to consider three different scenarios for ASL thickness. As ASL thickness cannot be measured *in vivo*, we used a number of ASL thicknesses in our model that covered the entire range found previously in *in vitro* data from CF bronchial epithelial cultures [28]. We also did not take into account dissolution of the inhaled antibiotic in sputum that can cover the airway epithelia [42]. Although mucus layer thickness can vary between patients and throughout the bronchial tree, it is reasonable to assume that this ASL layer will be at least 3 μm (thinnest ASL of CF epithelia found *in vitro*). Thus, it is likely that the concentrations we computed are too optimistic. Areas covered by mucus, especially those in regions of the lung with severe disease, may have even lower antibiotic concentrations, potentially decreasing below MIC_90_.

While ASL concentration is generally considered to be a reliable marker of alveolar antibiotic concentration [39–41], it is likely only an approximation as it relies on several assumptions. This model does not take into account drug uptake by alveolar macrophages as a measure of intracellular penetration in the lungs [43]. Especially in the chronically infected lung, macrophages may play a substantial role in the pharmacodynamics of anti-infective agents. This model also does not take into account binding of AZLI to sputum [43]. Only unbound drug concentrations are considered to be microbiologically active. In a single study, it was observed that there was little binding of AZLI to CF sputum [30]. Therefore it is not likely that this effect has a substantial effect on our data. We did not account for mucociliary and cough clearance, which further reduces AZLI concentrations in the airways, and this occurs within minutes after inhalation [44]. Our model assumes that the microbiological effect of a β-lactam antibiotic, such as AZLI, is best predicted using function of time above the MIC (T>MIC) [45, 46]. Unfortunately, the half-life of AZLI in the airways is not precisely known, but is thought to be approximately two hours in serum. Therefore, a prediction of efficacy of AZLI in the airways based on a single time-point immediately after inhalation is an approximation only. Thus, even though we calculated that the AZLI concentration was well above MIC_90_ for most simulated conditions immediately after nebulization, concentrations may decrease well below MIC_90_, especially in diseased areas, before a new dose of AZLI is nebulized.

We estimated the concentration of AZLI that could be considered effective for killing *Pa* strains based on previously reported data. However, the ideal AZLI concentration for effective killing of *Pa in vivo* is not well-defined, and varies largely between studies, with MIC_90_ values ranging between 32 and 128 μg/ml [29, 30, 47–49]. Terms indicating the efficacy level of antibiotics, e.g. MIC and MIC_90_, have been used interchangeably in other studies, making comparison difficult [45, 46]. For AZLI, most susceptible bacteria are killed at concentrations 1 to 4-fold their MIC. However, antibiotic concentrations required for killing *Pa* strains in biofilms are substantially higher than for killing *Pa* strains in planktonic growth. In an *in vitro* biofilm model, the time-dependent killing pattern of ceftazidime and imipenem in planktonic bacteria was changed to concentration-dependent killing for biofilm cells. Because of this, higher doses and longer treatment times with ceftazidime were required for the biofilm-growing *Pa* than for planktonic cells. While a concentration of 128xMIC was bactericidal for the wild-type strain (PAO1), a concentration of 2048xMIC was required for its ß-lactamase overproducing mutant (PAΔDDh2Dh3) [50]. In the registration studies of AZLI, concentrations of more than 2048 μg/ml were required to achieve bactericidal killing of *Pa* in some cases [48, 49, 51]. This corresponds to an AZLI concentration of more than 16-fold the MIC_90_ referred to in this study. Studies on the efficacy of AZLI mostly use a threshold of 10-fold MIC_90_, but without clear explanation [29, 30]. This threshold was also used for our analyses in this manuscript, but no claims can be made concerning its clinical significance.

Airways with a diameter below 1–2 mm could not be reconstructed from CT data, and were added to the model using Phalen’s description of the airway tree in infants, children and adolescents [12]. These model data are derived from subjects without lung disease and were combined with the assumption of homogenous aerosol distribution in these small airways. In CF, it is well recognized that the small airways are progressively involved in early life lung disease [3, 4]. Hence, it is likely that our model underestimates the heterogeneity of aerosol deposition, with the assumption of normal structure of the small airways. Moreover, the small airways might be most prone to *Pa* infection [9] and hence an inhibited AZLI delivery might be most unfavorable for this part of the lung. Even though we could not use real data for the simulation of small airways, the results are still highly relevant for clinical practice. The knowledge that lobes with substantial structural damage receive less inhaled antibiotic has consequences for the current standard treatment regimen. Current therapy is “one size fits all”: patients from all ages and with a variety of disease severities are treated with the same regimen. It might well be that a higher dose is needed for patients with more advanced disease to achieve antibiotic concentrations above MIC in all airway generations. Because structural lung disease severity is generally known by the treating physician, personalized therapy could be used, for example, by increasing antibiotic dose, in patients who do not respond to standard treatment.

We did not use patient-specific breathing profiles and upper airway models for this study, since they were not available. Clearly, this would have improved the precision of the simulations. However, as we covered a wide range of breathing patterns in our model, we believe that this represents a significant limitation in our work.

## Conclusions

We demonstrated that inhaled antibiotic concentrations in the small airways are highly patient-specific. A clear relation was found between patient-specific severity of localized lung disease and antibiotic concentrations throughout the lung. This method opens up the possibility of personalizing inhaled antibiotic dose to improve treatment efficacy.

## Supporting Information

S1 Fig Breathing profiles (High—Average—Low). Inhalation part of breathing profiles. Low breathing profile: tidal volume of 6 ml/kg (228 ml) and respiration rate of 14 breaths per minute. Average breathing profile: tidal volume of 10 ml/kg (380 ml) and respiration rate of 18 breaths per minute. High breathing profile: tidal volume of 14 ml/kg (532 ml) and respiration rate of 22 breaths per minute.
(TIF)

S2 Fig Influence of increases in aerosol diameter and increases in airway surface liquid thickness on AZLI concentrations. AZLI concentrations per lobe showing the influence of increases in aerosol diameter and increases in airway surface liquid (ASL) thickness on the AZLI concentrations. Data are presented as median (range). In each figure the AZLI concentrations are shown per separate lung lobe for the 3 different sizes of aerosol diameter. White bars represent the smallest aerosol diameter (2.9 μm), light grey bars represent the median aerosol diameter (3.18 μm) and dark grey bars represent the largest aerosol diameter (4.35 μm). The separate parts of the figure represent the 3 different thicknesses of ASL. Part S2a shows the AZLI concentrations calculated for the thin ASL (3 μm), part S2b shows the AZLI concentrations calculated for the median ASL (5 μm) and part S2c shows the AZLI concentrations calculated for the thick ASL (7 μm). RUL = right upper lobe, RML = right middle lobe. RLL = right lower lobe, LUL = left upper lobe, LLL = left lower lobe. The larger the aerosol diameter and the thicker the ASL the lower were the AZLI concentrations. Note differences in the scale on the vertical axes between the 3 separate parts of the figure.
(TIF)

S1 Table P-values of pairwise comparison of AZLI concentrations between lobes, accompanying Fig. 3. P-values of comparison between lobes in AZLI concentrations for the scenario of thick lining fluid with largest aerosol diameter. P-values in bold represent significant differences. RUL = right upper lobe, RML = right middle lobe. RLL = right lower lobe, LUL = left upper lobe, LLL = left lower lobe.
(DOCX)

S1 Text Supplementary Methods. (DOC)
