# Supplementary figures and images for: Patient-Specific Modeling of Regional Antibiotic Concentration Levels in Airways of Patients with Cystic Fibrosis: Are We Dosing High Enough?
Source: PLoS One. 2015 Mar 3;10(3):e0118454. doi: 10.1371/journal.pone.0118454 (PMC4348481; doi:10.1371/journal.pone.0118454)

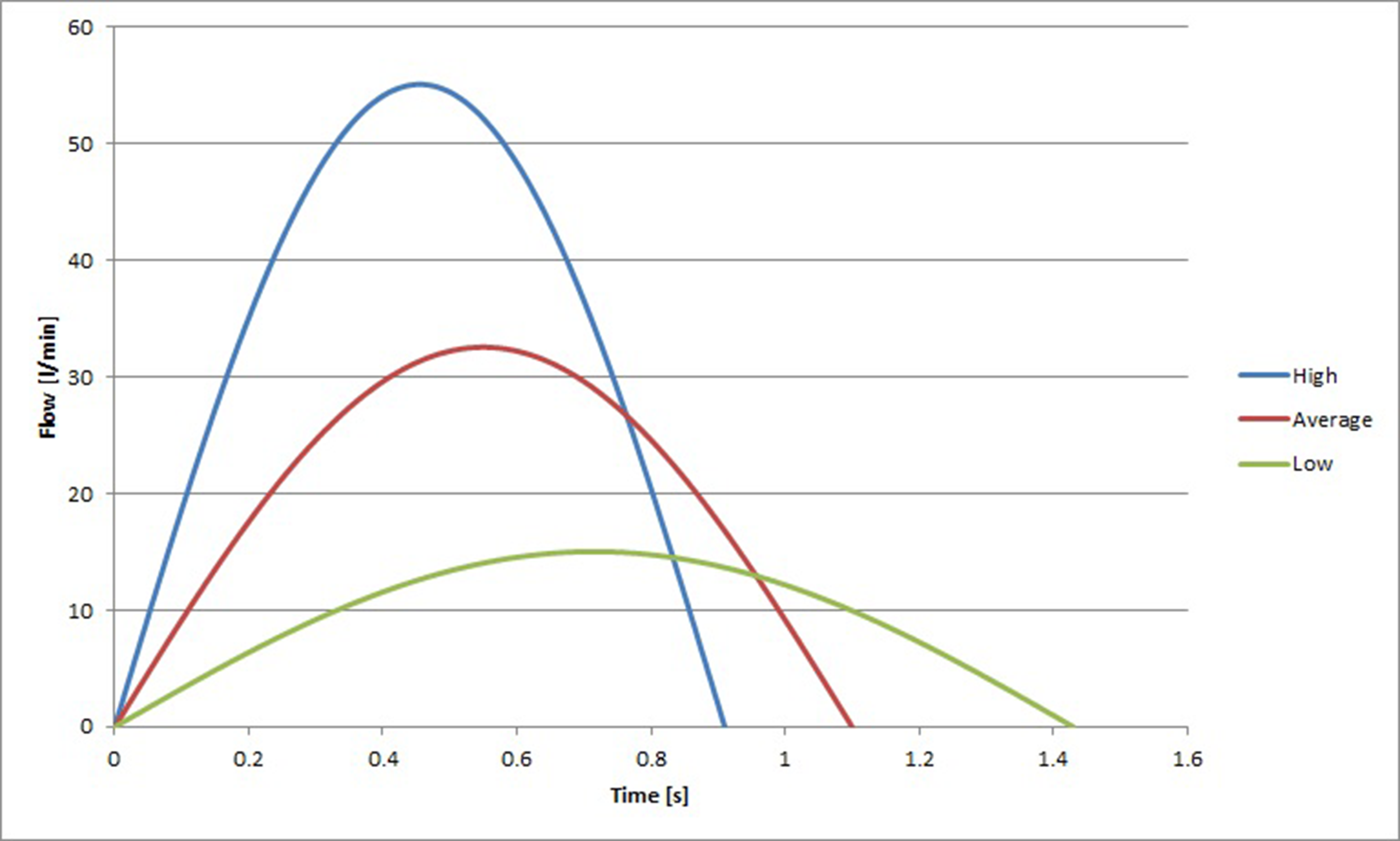

Supplement: S1 Fig — Inhalation part of breathing profiles. Low breathing profile: tidal volume of 6 ml/kg (228 ml) and respiration rate of 14 breaths per minute. Average breathing profile: tidal volume of 10 ml/kg (380 ml) and respiration rate of 18 breaths per minute. High breathing profile: tidal volume of 14 ml/kg (532 ml) and respiration rate of 22 breaths per minute. (TIF) [file pone.0118454.s001.tif]

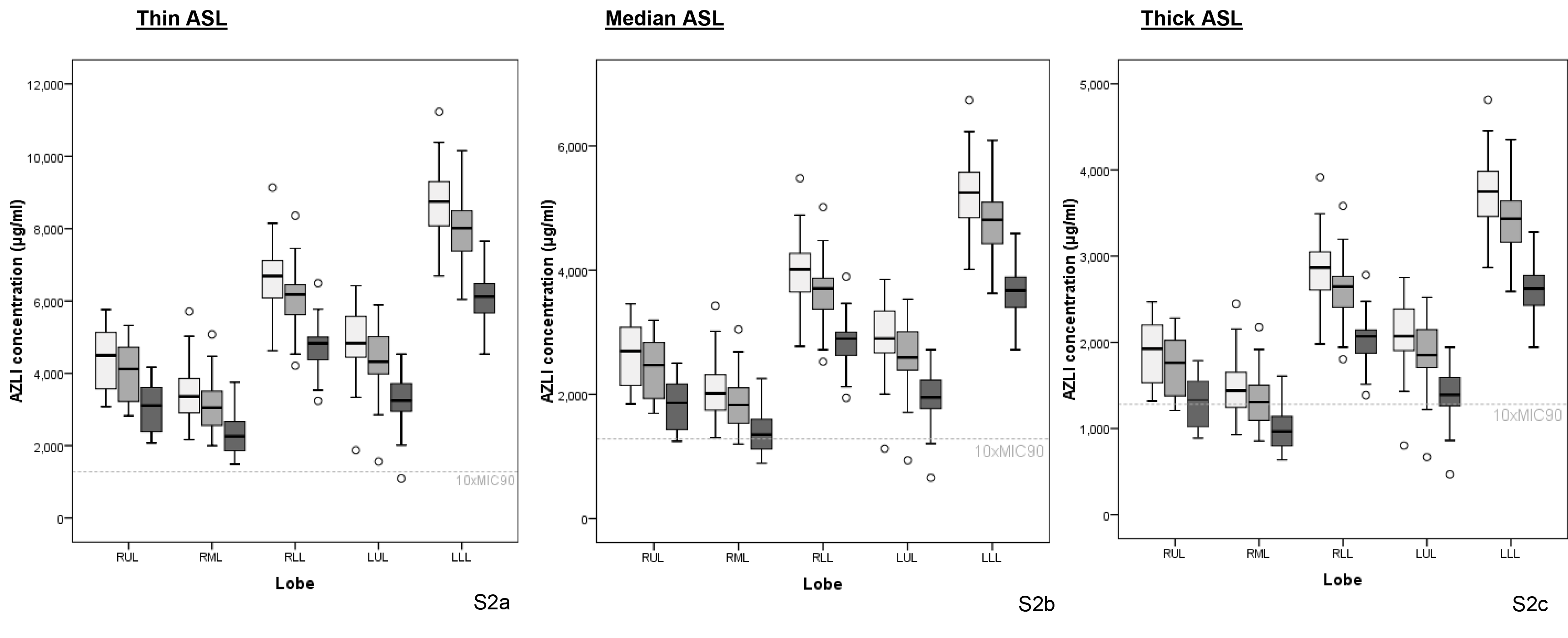

Supplement: S2 Fig — AZLI concentrations per lobe showing the influence of increases in aerosol diameter and increases in airway surface liquid (ASL) thickness on the AZLI concentrations. Data are presented as median (range). In each figure the AZLI concentrations are shown per separate lung lobe for the 3 different sizes of aerosol diameter. White bars represent the smallest aerosol diameter (2.9 μm), light grey bars represent the median aerosol diameter (3.18 μm) and dark grey bars represent the largest aerosol diameter (4.35 μm). The separate parts of the figure represent the 3 different thicknesses of ASL. Part S2a shows the AZLI concentrations calculated for the thin ASL (3 μm), part S2b shows the AZLI concentrations calculated for the median ASL (5 μm) and part S2c shows the AZLI concentrations calculated for the thick ASL (7 μm). RUL = right upper lobe, RML = right middle lobe. RLL = right lower lobe, LUL = left upper lobe, LLL = left lower lobe. The larger the aerosol diameter and the thicker the ASL the lower were the AZLI concentrations. Note differences in the scale on the vertical axes between the 3 separate parts of the figure. (TIF) [file pone.0118454.s002.tif]
