# Supplementary material for: Patient-Specific Modeling of Regional Antibiotic Concentration Levels in Airways of Patients with Cystic Fibrosis: Are We Dosing High Enough?
Source: PLoS One. 2015 Mar 3;10(3):e0118454. doi: 10.1371/journal.pone.0118454 (PMC4348481; doi:10.1371/journal.pone.0118454)
Supplement: S1 Table — P-values of comparison between lobes in AZLI concentrations for the scenario of thick lining fluid with largest aerosol diameter. P-values in bold represent significant differences. RUL = right upper lobe, RML = right middle lobe. RLL = right lower lobe, LUL = left upper lobe, LLL = left lower lobe. (DOCX) [file pone.0118454.s003.docx]

**Table S1. P-values of pairwise comparison of AZLI concentrations between lobes, accompanying figure 3.**

| **Lobe** | **Comparison with other lobes**  **(p-values)** | | | | |
| --- | --- | --- | --- | --- | --- |
|  | **RUL** | **RML** | **RLL** | **LUL** | **LLL** |
| RUL | - | **0.002** | **1.4E-09** | 0.156 | **2.7E-10** |
| RML | **0.002** | - | **2.7E-10** | **5.6E-06** | **2.7E-10** |
| RLL | **1.4E-09** | **2.7E-10** | - | **6.8E-11** | **5.3E-10** |
| LUL | 0.156 | **5.6E-06** | **6.8E-11** | - | **3.3E-13** |
| LLL | **2.7E-10** | **2.7E-10** | **5.3E-10** | **3.3E-13** | - |

P-values of comparison between lobes in AZLI concentrations for the scenario of thick lining fluid with largest aerosol diameter. P-values in bold represent significant differences. RUL = right upper lobe, RML = right middle lobe. RLL = right lower lobe, LUL = left upper lobe, LLL = left lower lobe.
