# Supplementary material for: Patient-Specific Modeling of Regional Antibiotic Concentration Levels in Airways of Patients with Cystic Fibrosis: Are We Dosing High Enough?
Source: PLoS One. 2015 Mar 3;10(3):e0118454. doi: 10.1371/journal.pone.0118454 (PMC4348481; doi:10.1371/journal.pone.0118454)
Supplement: S1 Text — (DOC) [file pone.0118454.s004.doc]

# Supplementary Methods

## Chest computed tomography

All routine chest CT scan sets were acquired using a 128-slice CT scanner (Somatom Definition Flash; Siemens, Erlangen, Germany). Thirty minutes prior to performing the CT scan, a lung function technician trained the study subjects by practicing the required spirometry manoeuvres in the supine position. Children were trained to obtain a breath-hold at maximal inspiration (total lung capacity, TLC) and maximal expiration (residual volume, RV) for 5–15 seconds. The inspiratory and expiratory slow vital capacities (SVC) achieved during the training were used as the reference values for the spirometric results during the CT scan. The reference SVCs were performed according to the ATS/ERS criteria[3]. Breathing instructions during the CT scan were given by the same lung function technician. During the scan, the lung technician monitored in real time the inspired and expired volumes on the computer screen of the CT-compatible spirometer setup. When the patient reached the correct TLC (inspiratory scan) or RV (expiratory scan) breath hold level, the lung function technician signalled the CT-technician to start scanning. For the technician-guided technique the same breathing instructions were given during the CT scan; however, the inspired and expired volumes were not spirometrically measured.

CT settings
Tube voltages of 80kV (patients < 35kg) or 110kV (patients ≥ 35kg) were used with a 0.6s rotation time. Scanning was done from apex of the lung to base at 1.5 pitch and 6x2mm collimation. Images were reconstructed with a slice thickness ≤ 1.0mm, a slice increment ≤ 0.6mm and kernel B75f. For the inspiratory protocol, a modulating current was used (Siemens) with a reference tube current-time product of 20mAs for optimal image quality. For expiratory CTs, a tube current fixed at 25mA with an effective tube current-time product of 10 mAs (the typical value for a 5-year-old child) was used, producing a lower radiation dose than the inspiratory protocol with sufficient image quality. Total radiation dose was in the order of 0.75 mSv for children below the age of 6 years and 1 mSv in older children.

## CT evaluation

To quantify chest CT abnormalities, we used the validated CF-CT scoring system [4]. This scoring method evaluates the 5 lung lobes and the lingula as a sixth lobe for the following components: 1) severity and extent of central and peripheral bronchiectasis; 2) severity and extent of central and peripheral airway wall thickening; 3) extent of central and peripheral mucus plugging; 4) extent of opacities (atelectasis, consolidation, ground glass pattern); 5) extent of cysts and bullae on inspiratory CTs and 6) the pattern and extent of trapped air on expiratory CTs. The maximal possible composite CT score is 207 points. In the CF-CT scoring method, the CF-CT composite score is calculated by summing the component scores per lobe. Instead of using the CF-CT composite score, the component scores per lobe were used for analysis. The lobar specific component scores were expressed as a percentage of the maximum possible component score per lobe. The component scores for bronchiectasis, airway wall thickening and air trapping were used for analysis.

Prior to scoring, all CT scans were de-identified (Myrian®; Intrasense, Montpelier, France). Next, scans were scored in random order by an experienced observer, with more than 2 years’ experience in scoring, who was blinded to clinical background. To assess inter-observer agreement, a second observer with 4 months scoring experience rescored all CT scans. Both observers were initially trained in CF-CT scoring using a standardized instruction module and training sets. Good intra- and inter-observer agreement was established on the training sets before scoring the study CT scans. To establish the intra-observer agreement, observer 1 rescored 25 random selected scans after 3 months. CF-CT scores of observer 1 were used for analysis.

# References Supporting Information

1. Stanojevic S, Wade A, Stocks J, Hankinson J, Coates AL, et al. (2008) Reference ranges for spirometry across all ages: A new approach. Am J Respir Crit Care Med 177: 253-260.

2. Zapletal A, Naidr J, Pohunek P. (1992) A brief description of methods for studying pulmonary function in children and adolescents. Cesk Pediatr 47: 520-523.

3. Miller MR, Hankinson J, Brusasco V, Burgos F, Casaburi R, et al. (2005) Standardisation of spirometry. Eur Respir J 26: 319-338.

4. Wainwright CE, Vidmar S, Armstrong DS, Byrnes CA, Carlin JB, et al. (2011) Effect of bronchoalveolar lavage-directed therapy on pseudomonas aeruginosa infection and structural lung injury in children with cystic fibrosis: A randomized trial. JAMA 306: 163-171.

5. Talma H. (2011) Groeidiagrammen 2010: Handleiding bij het meten en wegen van kinderen en het invullen van groeidiagrammen. : [S.I.] : TNO innovation for life.

6. Wallis LA, Healy M, Undy MB, Maconochie I. (2005) Age related reference ranges for respiration rate and heart rate from 4 to 16 years. Arch Dis Child 90: 1117-1121.

7. Phalen RF, Oldham MJ, Kleinman MT, Crocker TT. (1988) Tracheobronchial deposition predictions for infants, children and adolescents. Ann  occup  Hyg 32: 11-21.

8. Tarran R, Button B, Picher M, Paradiso AM, Ribeiro CM, et al. (2005) Normal and cystic fibrosis airway surface liquid homeostasis. the effects of phasic shear stress and viral infections. J Biol Chem 280: 35751-35759.

9. Tarran R, Button B, Boucher RC. (2006) Regulation of normal and cystic fibrosis airway surface liquid volume by phasic shear stress. Annu Rev Physiol 68: 543-561.

10. Retsch-Bogart GZ, Quittner AL, Gibson RL, Oermann CM, McCoy KS, et al. (2009) Efficacy and safety of inhaled aztreonam lysine for airway pseudomonas in cystic fibrosis. Chest 135: 1223-1232.

11. King P, Lomovskaya O, Griffith DC, Burns JL, Dudley MN. (2010) In vitro pharmacodynamics of levofloxacin and other aerosolized antibiotics under multiple conditions relevant to chronic pulmonary infection in cystic fibrosis. Antimicrob Agents Chemother 54: 143-148.

12. Oermann CM, Retsch-Bogart GZ, Quittner AL, Gibson RL, McCoy KS, et al. (2010) An 18-month study of the safety and efficacy of repeated courses of inhaled aztreonam lysine in cystic fibrosis. Pediatr Pulmonol 45: 1121-1134.

13. Bland JM, Altman DG. (1986) Statistical methods for assessing agreement between two methods of clinical measurement. Lancet 1: 307-310.

14. Benjamini Y, Hochberg Y. (1995) Controlling the false discovery rate: A practical and powerful approach to multiple testing. J R Statist Soc B 57: 189-300.
